# Prestin is an anion transporter dispensable for mechanical feedback amplification in *Drosophila* hearing

**DOI:** 10.1007/s00359-014-0960-9

**Published:** 2014-11-21

**Authors:** Ryan G. Kavlie, Janice L. Fritz, Florian Nies, Martin C. Göpfert, Dominik Oliver, Joerg T. Albert, Daniel F. Eberl

**Affiliations:** 1 The Ear Institute, University College London, 332 Gray’s Inn Road, London, WC1X 8EE UK; 2 Science, Technology, Engineering, and Math Division, St. Clair County Community College, Port Huron, MI 48060 USA; 3 Institute for Physiology and Pathophysiology, Philipps University, Marburg, Germany; 4 Department of Cellular Neurobiology, Georg-August-Universität Göttingen, Göttingen, Germany; 5 Department of Biology, University of Iowa, Iowa City, IA 52246 USA

**Keywords:** Prestin, Mechanical amplification of sound, Auditory amplification, Drosophila hearing, NompC

## Abstract

In mammals, the membrane-based protein Prestin confers unique electromotile properties to cochlear outer hair cells, which contribute to the cochlear amplifier. Like mammals, the ears of insects, such as those of *Drosophila melanogaster*, mechanically amplify sound stimuli and have also been reported to express Prestin homologs. To determine whether the *D. melanogaster* Prestin homolog (*dpres*) is required for auditory amplification, we generated and analyzed *dpres* mutant flies. We found that *dpres* is robustly expressed in the fly’s antennal ear. However, *dpres* mutant flies show normal auditory nerve responses, and intact non-linear amplification. Thus we conclude that, in *D. melanogaster*, auditory amplification is independent of Prestin. This finding resonates with prior phylogenetic analyses, which suggest that the derived motor function of mammalian Prestin replaced, or amended, an ancestral transport function. Indeed, we show that *dpres* encodes a functional anion transporter. Interestingly, the acquired new motor function in the phylogenetic lineage leading to birds and mammals coincides with loss of the mechanotransducer channel NompC (=TRPN1), which has been shown to be required for auditory amplification in flies. The advent of Prestin (or loss of NompC, respectively) may thus mark an evolutionary transition from a transducer-based to a Prestin-based mechanism of auditory amplification.

**Electronic supplementary material:**

The online version of this article (doi:10.1007/s00359-014-0960-9) contains supplementary material, which is available to authorized users.

## Introduction

Hearing is a physiologically delicate process. The sensitivity of vertebrate ears results from the intricate and unique molecular transduction machinery that resides in the hair bundles of their sensory hair cells. Within the bundles, the interplay between force-gated transducer channels and adaptation motors generates forces that amplify sound-induced bundle movements and thereby boost vertebrate auditory performance while in mammals, an additional amplification mechanism is provided by a Prestin-mediated electromotile activity (Hudspeth 2008). Understanding the molecular, and mechanistic, details of auditory transduction is essential for understanding the bases of hearing and the causes of deafness.

In mammals, the exquisite sensitivity and frequency selectivity is achieved through an active amplification of sound-evoked vibrations of the basilar membrane (BM) within the cochlea. BM vibrations deflect specialized microvilli, or stereocilia, of inner ear hair cells and thereby directly open mechano-electric transducer channels (Fettiplace and Hackney 2006; Peng et al. 2011). Two types of auditory hair cells exist, both of which harbor mechanosensory transducer channels: (1) inner hair cells (IHCs) represent the ‘sensory arm’ of cochlear function and send the auditory information, created by the BM vibrations, to the brain; (2) outer hair cells (OHCs) represent the ‘motor arm’ of cochlear function and respond to transduction-induced changes in membrane potential by contractions and expansions of their cell bodies, thereby amplifying the BM vibrations. The molecular motor responsible for this electromotility of OHCs is Prestin, a member of the SLC26 anion transporter family (Dallos and Fakler 2002). Loss of Prestin in mice raises the threshold at which they respond to sound, and a mutation in Prestin may cause hereditary hearing loss in humans (Liberman et al. 2002; Liu et al. 2003).

Like vertebrate hair cells, the auditory neurons of the fruit fly *D. melanogaster* are endowed with an active, force-generating process that involves mechanically gated transducer channels and adaptation motors acting in concert to boost hearing (Göpfert et al. 2005; Nadrowski et al. 2008). The macroscopic performance of the entire *D. melanogaster* ear is governed by the properties of these active transducer modules, and the fly’s external antennal sound receiver can be used to directly probe auditory transducer function in vivo in both wild-type and mutant flies (Albert et al. 2007; Kamikouchi et al. 2010).

*D. melanogaster* responds to sound through the Johnston’s organ (JO), which is made up of an array of ciliated neurons in function analogous to the IHCs of the mammalian cochlea (Eberl 1999). The motor mechanism by which sound-induced vibrations are actively amplified by the JO is unknown but the origin of this amplification is attributable to JO neurons (Göpfert et al. 2005). JO does not have OHCs to amplify vibrations. Instead, the dendritic cilia of JO neurons may contract to boost the vibratory response to faint sounds (Göpfert and Robert 2002; Kernan 2007; Lee et al. 2008). A transducer-based, quantitative model, which assumes that (as yet molecularly unknown) motor proteins act in series with the actual transducer channels, was found to explain the mechanical and electrical responses of the fly’s ear to small disturbances (Nadrowski et al. 2008). An alternative hypothesis as to how JO neurons might amplify sound is through a Prestin-based electromotility. In this hypothesis, the *D. melanogaster homolog* Prestin (dpres) may contribute to ciliary motility by causing contraction in the membranes of the JO neurons.

The possibility that dpres contributes to transducer-based active amplification of the sound-induced vibrations has escaped rigorous molecular evolutionary analysis of Prestin orthologs in representative species which suggested that Prestin-based electromotility exists only in mammals (Okoruwa et al. 2008; Tan et al. 2011). In fact, specific residues have been identified as being essential for Prestin’s evolution from an anion transporter to a protein with electromotility responsible for OHC electromotility necessary for sound amplification (Schaechinger et al. 2011; Tan et al. 2011). New research, however, questions the time at which this electromotility evolved in metazoans. While an early study in chickens revealed no Prestin-based electromotility (He et al. 2003), a more recent study showed Prestin-like signatures in chicken short hair cell stiffness (orthologous function to mammalian outer hair cells), active amplification and frequency tuning of the auditory organ (Beurg et al. 2013). This suggests that the amplifying function of Prestin could be more highly conserved than previously supposed.

The goal of this study was to determine whether dpres is necessary for non-linear mechanical amplification in the hearing organ of *D. melanogaster*. This was accomplished by examining the expression pattern of *dpres*, and by analyzing the phenotypes of newly generated *dpres* loss-of-function mutants. The results demonstrate that *dpres* is expressed in JO neurons yet is not required for auditory amplification in *D. melanogaster*. Instead, dpres function may be restricted to the role as anion antiporter, consistent with previous reports (Hirata et al. 2012a, b).

## Methods

### Fly lines

The *D. melanogaster* strains carrying *w* ^*1118*^, *dpres*-Gal4, and *dpres* mutants (engineered from homologous recombination and P-element mobilization described below) were used in the analysis. All flies were raised at 25 °C and 60 % humidity.

### Expression analysis

The 5′ regulatory sequences of *dpres* were cloned into the pPTGal vector (Sharma et al. 2002) and injected into *w* ^*1118*^ embryos to produce a transgenic line. The resulting line was crossed to a UAS-EGFP reporter to determine the d*pres* expression pattern.

### Homologous recombination

**Fig. 1:**
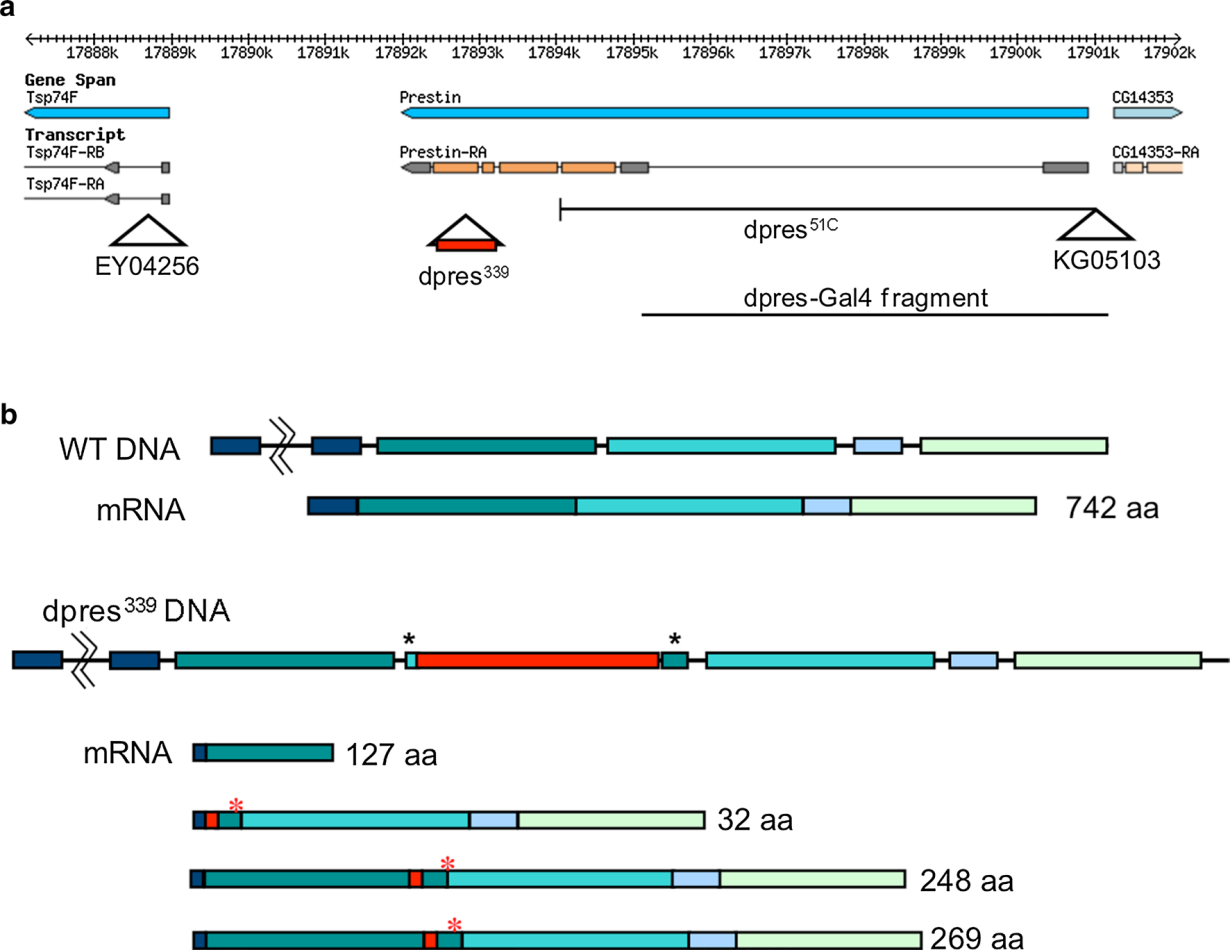
Generation of mutations in *dpres*. **a** The genomic organization of the *dpres* gene on the left arm of chromosome 3 at polytene chromosome position 75A9. Gene annotations (*blue*) are shown below the sequence coordinates (*top line*) of 3L, with flanking genes Tsp74F and CG14353. The *dpres* gene (shown as Prestin) is encoded on the minus strand with the transcript structure shown below (coding exons in *orange*, untranslated regions in *gray*). The sites of P-element insertions EY04256 and KG05103 are shown as *triangles*, and extent of the *dpres* ^*51C*^ deletion derived from an imprecise excision of KG05103 is shown by the labeled *black line*. The location of the lesion in homologous recombination allele *dpres* ^*339*^ is shown as a triangle with the *w* ^*hs*^ insertion (*red box*). The extent of the enhancer fragment used in generating the *dpres*-Gal4 line is also depicted. The upper part of the panel was obtained from GBrowse, accessed through Flybase. **b** Transcript structure of *dpres* in wild type and homologous recombination mutant. The wild-type transcript contains all four coding exons (*colored*) and encodes a 742 amino acid protein. Ends-out homologous recombination generated an insertion of the *w* ^*hs*^ element (providing w^+^ eye color), with flanking duplications of the 3′ end of exon 3 and the 5′ end of exon 3 (*black asterisks*) containing engineered stop codons. RT-PCR amplified several *dpres* transcripts, none of which are wild type. Splicing past the *w* ^*hs*^ insert causes a frame shift leading to tandem stop codons near the 3′ end of exon 2 (*red asterisks*)

We used an ends-out targeting approach using the homologous recombination techniques in *D. melanogaster* (Rong and Golic 2000, 2001). First, PCR-based site-directed mutagenesis using mutant primers was used to engineer two in-frame stop codons each in the first and third exons of the *dpres* gene (Fig. 1b, asterisks; Fig. S1). An* Sce*I cut site was created by overlapping oligonucleotides and inserted into a* Bam*HI site between the engineered mutations, approximately 500 bp from each to avoid loss of the mutations due to degradation from the cut site (Rong et al. 2002). The mutant *dpres* construct was ligated into the pTV2 NotI plasmid. pTV2 contains the sequence necessary for insertion of the P-element into the genome, FRT sites for excision of the circular donor DNA molecule, and a modified *w* ^+^ gene (*w* ^*hs*^) for tracking of the insertion.

The construct was injected into fly embryos to make a transgenic line that was then crossed to FLP,* Sce*I for targeted mutagenesis. Transgenic larvae were heat-shocked at 37 °C for 30 min to produce a circularized DNA element that recombined with *dpres*. Stable lines were produced from the progeny and screened using PCR to identify lines with a disrupted *dpres* gene. Line 339 was confirmed as a *dpres* mutant by long-template PCR and therefore renamed *dpres* ^*339*^.

### P-element mobilization

A P-element transposase line [Dr, P(Δ2–3)] was crossed to the P-element insertion lines KG05103 and EY04256 to generate EY04256 Dr, P(Δ2–3/KG05103 dysgenic flies, which were then crossed to CxD/TM3. Offspring were collected to create stable lines that were screened by PCR to identify imprecise excisions that removed *dpres*. Of the 101 balanced lines isolated, 92 were homozygous viable. Using PCR on 72 viable or lethal lines, we determined that one viable line, 51C, was a *dpres* deficiency spanning from the KG05103 insertion site past exon two (Fig. 1).

### RT-PCR

RT-PCR primers were designed against the *dpres* gene. PCR was performed on mRNA from late-stage embryos, larvae and adults to determine expression pattern of *dpres* mRNA from controls and homologous recombinants.

### Electrophysiology

Electrophysiology was performed on the antenna with methods described by Eberl et al. (2000). Briefly, the fly was immobilized in a pipette tip trimmed to allow the head to protrude. The pulse song, a component of the fly’s courtship song, was played on a loudspeaker and delivered to the fly through Tygon tubing to preserve near-field sound properties. The sound stimulus intensity was measured at 5.3 mm/s at the position of the antennae, using a calibrated Emkay NR3158 particle velocity microphone (Knowles). One electrolytically sharpened tungsten electrode was inserted in the junction between the first and second antennal segments while a second electrode penetrated the dorsal head cuticle as a reference. The signals were subtracted with a DAM50 differential amplifier (World Precision Instruments) and then digitized and normalized using a virtual instrument designed with Superscope 3.0 (G. W. Instruments) software. Responses to ten presentations of the stimulus were averaged, and the peak amplitudes of sound-evoked potentials (SEPs) were measured.

### Confocal microscopy

Confocal microscopy was performed using a Zeiss LSM510 microscope. Whole mount flies were prepared by immersing in 50 % glycerol and mounting on depression slides. Antennae were visualized by dissection and staining with rhodamine phalloidin.

### Laser Doppler vibrometry

Adult *D. melanogaster* aged 1–5 days after eclosion were mounted on a Teflon rod and immobilized using dental glue. A laser Doppler vibrometer (LDV; PSV-400 with an OFV-70 close-up unit and a DD-5000 displacement decoder, Polytec GmbH, Waldbronn, Germany) was used to record the oscillations of the arista. Signals were generated and recorded in the PSV 8.6 Scanning Vibrometer Software (GmbH, Waldbronn, Germany) and later processed in Sigmaplot (Systat Software Inc) to extract best frequencies and energy gain (more details in Göpfert et al. 2005). Mutant and control data were tested for normality (Shapiro–Wilk) and equal variance (rejection thresholds set to *p* < 0.05 in both cases) prior to further statistical analysis. All data presented here passed both the normality and equal variance test and were analyzed using a two-tailed *t* test.

### Anion transport measurements

*A dpres*-GFP fusion construct (in pEGFP-N2 vector) was transfected into CHO cells using JetPEI transfection reagent (Polyplus). Patch-clamp recordings of electrogenic transport currents were performed as described (Schaechinger and Oliver 2007; Schaechinger et al. 2011). Briefly, cells with unequivocal membrane fluorescence were selected (24–48 h after transfection). Whole-cell patch-clamp recordings were carried out at room temperature (20–22 °C) with an EPC10 amplifier (HEKA electronics, Lambrecht, Germany) controlled by Patchmaster software (HEKA). Electrogenic anion transport was measured as the ionic transport current in response to command voltage ramps (−100 to +100 mV; 300 ms). Background currents recorded before application of extracellular transport substrates were subtracted from current traces. For comparison between cells, current densities were calculated by normalizing to whole-cell capacitance as a measure of membrane area.

Patch electrodes were filled with intracellular solution containing 160 mM CsCl, 1 mM HEPES, 1 mM K_2_EGTA, adjusted to pH 7.3 with KOH. Extracellular transport substrates were locally applied to the cells via a gravity-fed application capillary. Extracellular solution contained 10 mM NaCl, 150 mM Na-gluconate, 10 mM HEPES, and 2 mM Mg-gluconate, adjusted to pH 7.4 with NaOH. For extracellular application of divalent anions, 10 mM SO_4_ or oxalate substituted for chloride. For application of bicarbonate, 25 mM HCO_3_^−^ was exchanged for equimolar Cl^−^ and the solution was equilibrated with 5 % CO_2_.

## Results

### *dpres* is expressed in auditory neurons

The *D. melanogaster* gene CG5845 encodes dpres (Fig. 1a) (Weber et al. 2003). *dpres* is located at polytene chromosome position 75A9 on chromosome arm 3L (Fig. 1a). The predicted gene product is a 742 amino acid protein with sulfate transporter domains that overall shares 18 % identity and 31 % similarity with mammalian Prestin (Fig. S1).

If dpres were functionally orthologous to human Prestin, acting as a piezo crystal-like motor that augments the ears’ mechanical responses to sound, we would expect *dpres* to be expressed in the neurons of JO, and possibly other chordotonal organs. Therefore, we first characterized the *dpres* expression pattern.

**Fig. 2:**
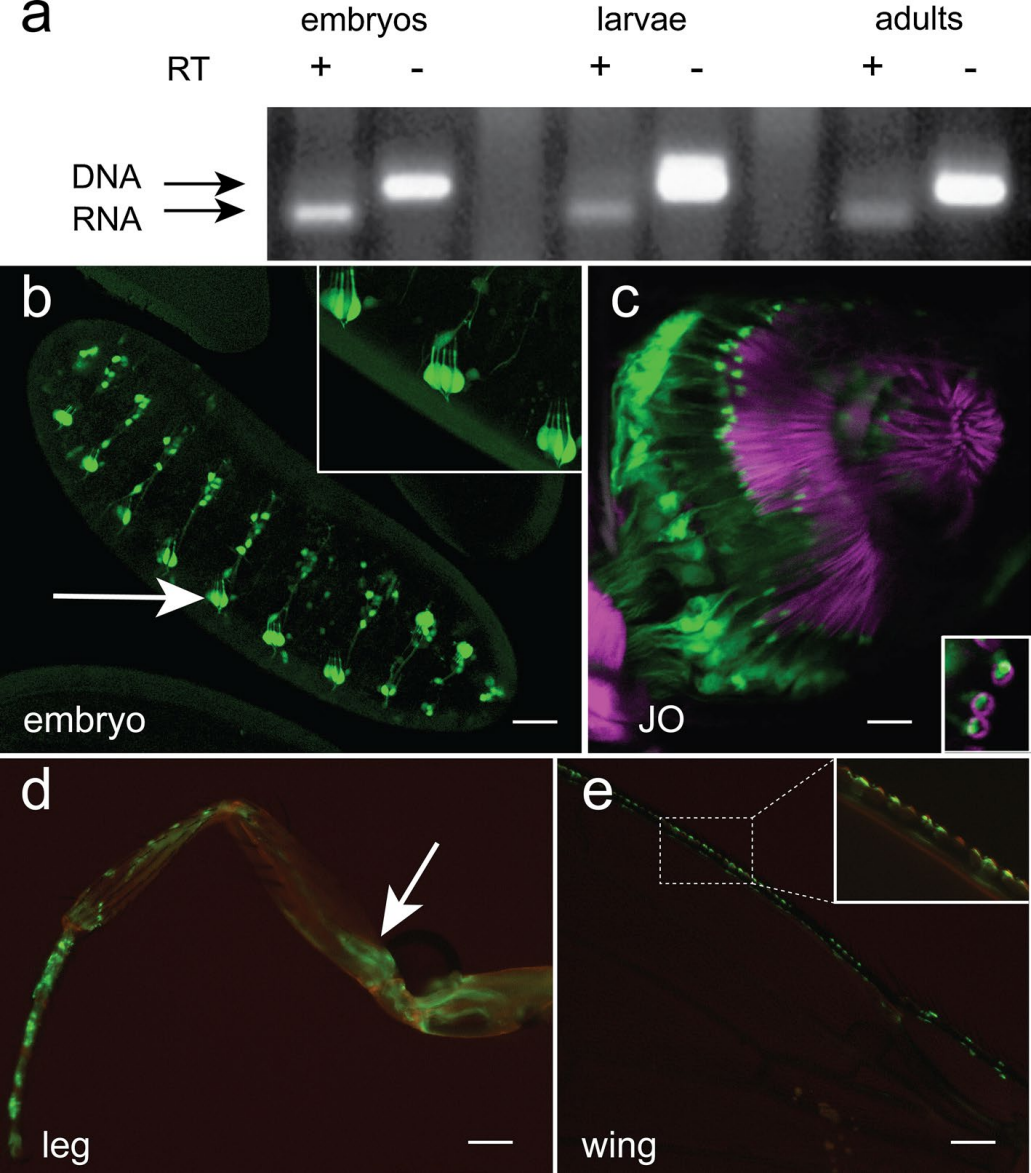
*dpres* promoter region drives expression in chordotonal organs. **a** RT-PCR amplifies dpres mRNA from embryos, larvae, and adults. Without reverse transcriptase (RT) the longer genomic DNA contamination amplifies, but with RT, the shorter spliced RNA product is distinguished. **b** A 6.1 kb fragment of the *dpres* regulatory region (shown in Fig. 1) connected to Gal4 drives expression of GFP in the peripheral nervous system of embryos. Expression is seen in the neurons of the embryonic lateral chordotonal organs (lch5′s) (*arrow* and inset). **c**–**e** *dpres*-Gal4 also drives strong expression in JO neurons (**c**) in the second antennal segment (*green*: GFP, magenta: rhodamine phalloidin) and in the femoral chordotonal organ of the leg (**d** *arrow*) and the sensory organs of the anterior wing margin (**e**). *Scale bars* represent 40 μm in (**b**), 10 μm in (**c**), and 100 μm in (**d**) and (**e**)

RT-PCR demonstrated expression in embryos, larvae and adults (Fig. 2a). To obtain evidence for *dpres* expression pattern, the 5′ regulatory region was cloned upstream of Gal4 using the P-element vector pPTGal (Sharma et al. 2002) to produce a *dpres*-Gal4 transgenic line (Fig. 1a). The *dpres* expression pattern was visualized by crossing the Gal4 line to a UAS-EGFP reporter. *dpres* is expressed in the chordotonal neurons of embryos and adults (Fig. 2b, c). *dpres* is also expressed in mechanosensitive neurons of the legs and wings indicating possible anion antiporter functions in these sensory organs as well (Fig. 2d, e). In particular, *dpres* is expressed in the neurons of the JO in the second antennal segment, which are the auditory mechanoreceptors (Eberl 1999).

### *dpres* mutants have no effect on SEPs

**Fig. 3:**
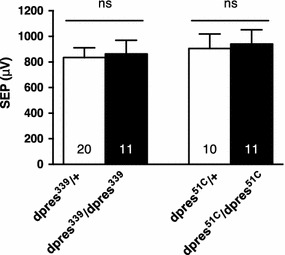
*dpres* mutants have no effect on auditory sound-evoked potentials. Average peak amplitudes of sound-evoked potentials (SEPs), extracellular potentials recorded from the vicinity of the antennal nerve in response to simulated pulse song. Two-tailed unpaired *t* tests showed no significant difference between the *dpres* mutants and their heterozygous controls, (*p* = 0.8295 for *dpres* ^*339*^, *p* = 0.8306 for *dpres* ^*51C*^). Numbers of antennae recorded are indicated at the bottom of each *bar*. *Error bars* indicate standard error of the mean (SEM)

Because *dpres* is the homolog of mammalian Prestin and is expressed in JO neurons, we expected reduced hearing in *dpres* mutants in response to sound stimuli. We took a reverse genetics approach to engineer mutations in the *dpres* gene. Homologous recombination was used to disrupt *dpres* with 5 kb insertion containing the *w* ^*hs*^ gene inserted in the middle of *dpres*. The recombination resulted in a small duplication of the 3′ end of exon two and the 5′ end of exon three, flanking the insert. RT-PCR amplified several *dpres* transcripts, none of which are wild type (Fig. 1b) and were very short or contained frameshifts to early stop codons. Electrophysiological recordings were conducted on the antennal nerve to determine whether sound-evoked potentials were affected in *dpres* mutant flies. SEPs from homozygous *dpres* ^*339*^/*dpres* ^*339*^ flies responded to the pulse stimulus and were indistinguishable from *dpres* ^*339*^/+ heterozygous sibling controls (Fig. 3).

To reconcile the lack of electrophysiological phenotype in the homologous recombinant allele *dpres* ^*339*^, we wondered whether the truncated proteins, up to 250 residues, from the aberrant transcripts could be partially functional. Therefore, we wanted to generate a deletion that completely removes the *dpres* coding sequences. To this end, we took two approaches. We first made use of the *w* ^*hs*^ element inserted into the homologous recombinant allele, providing w^+^ eye color, as a substrate for gamma-ray mutagenesis to select for w^−^ derivatives. Two w^−^ derivatives recovered were found to have cytologically visible deletions in the 75AB region of polytene chromosomes, and to form a lethal complementation group with *Df(3L)BSC8*. Because these deletions in all combinations removed other vital genes in addition to *dpres*, they were not useful for testing *dpres* function. In the second approach, we made use of two P-element insertions near *dpres* to generate imprecise excisions. To allow the possibility of recovering a synthetic deletion between these two elements by P-induced recombination, we generated flies containing both P-elements and the P-element transposase. From this we recovered *dpres* ^*51C*^, an imprecise excision of the KG05103 element with a deletion extending from the KG05103 insertion site into the *dpres* gene, thus removing the entire upstream regulatory region and the first two exons. As with *dpres* ^*339*^, we found that SEPs were unaffected in the *dpres* ^*51C*^ allele (Fig. 3).

### Mechanical amplification of sound is independent of *dpres* function

**Fig. 4:**
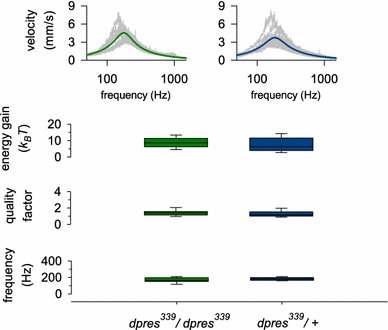
*dpres* mutants have normal mechanical amplification of sound. Free sound receiver fluctuations were recorded in *dpres* ^*339*^ mutants (*left*, *green*) and homozygous controls (*right*, *blue*). Shown are the FFT amplitudes of receiver velocities (*top*). No detectable difference was found in the receivers’ energy gain due to active mechanical amplification (two-tailed *t* test *p* = 0.357), the quality factor of the receivers’ mechanical tuning (two-tailed *t* test *p* = 0.439) and their best frequency (two-tailed *t* test *p* = 0.112) between *dpres* ^*339*^ mutants and the *dpres* ^*339*^/+ heterozygote control flies. The *boundaries* of the *boxes* closest to zero mark the 25th percentiles. The *lines* within the *boxes* *mark* the median, and the *boundaries* of the *boxes* farthest from zero mark the 75th percentiles. *Error bars* above and below the *box mark* the 90th and 10th percentiles, respectively. *n* = 13 for all values

To estimate the extent of mechanical feedback amplification in *dpres* loss-of-function mutations more directly, we analyzed the free fluctuations of unstimulated antennal sound receivers of mutants and control flies (see Göpfert et al. 2005 for details). In the receivers of wild-type flies, these recordings reflect two distinct phenomena: (1) the thermal energy of the environment and (2) the additional energy injected by JO neurons (Göpfert et al. 2005). Through their energy contributions, JO neurons cause characteristic shifts of both the frequency tuning and damping characteristics of the *D. melanogaster* antennal ear (Göpfert et al. 2005; Nadrowski and Göpfert 2009; Riabinina et al. 2011). A potential reduction (or loss) of mechanical feedback amplification in *dpres* mutants would thus result in predictable changes of the flies’ antennal mechanics. Our laser Doppler vibrometric analyses of the sound receivers’ free fluctuations (Fig. 4, top), however, showed no significant difference between *dpres* ^*339*^ */dpres* ^*339*^ homozygotes and *dpres* ^*339*^ */*+ control flies. Receiver best frequencies were 184 ± 18 Hz for control flies and 167 ± 31 Hz for mutant flies (two-tailed test: *p* = 0.112, *n* = 13, Fig. 4); a loss of amplification, for comparison, would have resulted in best frequency values of ~800 Hz [as for example seen in the ‘passive’ antennae of *tilB* null mutant flies (Göpfert et al. 2005)]. The values for the quality factor Q (a dimensionless, reciprocal measure of the antennae’s damping, with higher Q values indicating a less damped system) was 1.3 ± 0.4 in control flies and 1.4 ± 0.4 in mutants (*p* = 0.439, *n* = 13, Fig. 4). Auditory amplification in flies coincides with an undamping of the antennal receiver; a loss of amplification would have thus yielded lower *Q* values of ~0.8–1 (as characteristic of the passive antennal joint). Estimated energy gain values, finally, were 7.5 ± 4.1 k_B_T for controls and 8.8 ± 3.0 k_B_T for mutants (*p* = 0.357, *n* = 13, Fig. 4). A loss of amplification would have abolished all energy gain, leading to values of 0 k_B_T. Taken together these results demonstrate that *dpres* is not required for auditory feedback amplification.

**Fig. 5:**
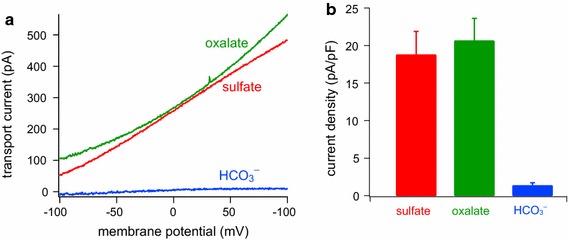
Electrogenic anion transport mediated by *dpres.* **a** Representative transport currents recorded from the same cell expressing *dpres*-GFP during subsequent applications of extracellular sulfate, oxalate (10 mM each) and HCO_3_^−^ (25 mM). Intra- and extracellular concentrations of Cl^−^ were 160 and 10 mM, respectively. Currents shown were recorded in response to voltage ramps (−100 to +100 mV) and background currents measured immediately before application of each transport substrate were subtracted. **b** Average currents at +80 mV from experiments as in (**a**), normalized to membrane surface. *Bars* represent means, *error bars* represent standard error, *n* = 10 cells, each tested sequentially for all three anions

The absence of a contribution to active mechanics rather suggested a function as an anion transporter. Vertebrate Prestin (SLC26A5) orthologs (Schaechinger and Oliver 2007; Gorbunov et al. 2014) and a closely related transporter from the ascidian *Ciona* *intestinalis* (Deng et al. 2013) are electrogenic anion antiporters that exchange sulfate and oxalate against chloride. We therefore explored the transport function in a heterologous expression system using whole-cell patch-clamp methods. When chloride was the only small cytoplasmic and extracellular anion, no currents above the background level of non-transfected CHO cells were observed, even in the presence of a chloride concentration gradient (not shown). Thus *dpres* does not mediate the chloride channel-like transport mode prevalent in the mammalian Prestin relatives SLC26A7 and SLC26A9 (Kim et al. 2005; Dorwart et al. 2007). However, as shown in Fig. 5, application of either sulfate or oxalate to the extracellular solution induced sizeable outward transport currents, consistent with stoichiometric exchange of the divalent anion against chloride, as in non-mammalian Prestin (Schaechinger and Oliver 2007). A recent study using *Xenopus* oocytes as the expression system suggested that dpres also mediates electrogenic exchange of bicarbonate versus chloride (Hirata et al. 2012b). We therefore attempted to measure transport currents with extracellular bicarbonate (25 mM) in the presence of an outward chloride gradient. In this situation, only negligible currents above background were observed (Fig. 5a, b), indicating that electrogenic exchange of n HCO_3_^−^ against 1 Cl^−^ occurs either at a low rate or is absent. Probably, *dpres* mediates electroneutral Cl^−^:HCO_3_^−^ exchange (i.e., with 1:1 stoichiometry), as found for the *C. intestinalis* Prestin homolog *Ci*-Slc26aα (Deng et al. 2013).

## Discussion

### The role of *dpres* in mechanical amplification of sound

Of the nine mammalian members of the SLC26 family, only Prestin is known to confer electromotility when transfected into mammalian cells (Zheng et al. 2000; Oliver et al. 2001; Lohi et al. 2002). Recent structural and functional analysis of Prestin suggests that the molecular mechanism underlying electromotility was derived evolutionarily from a primordial anion transport mechanism. Thus the protein domains that mediate anion transport, including a central anion binding site, are also critically involved in the electromotile function (Schaechinger et al. 2011; Gorbunov et al. 2014). Yet, by generating chimeric molecules between mammalian mechanically active Prestin and a non-mammalian transporter ortholog, it was shown that in principle, Prestin can mediate both anion transport and electromechanical activity at the same time (Schaechinger et al. 2011). It is thought that the movement of a voltage sensor, most likely related to the interaction of anions with the protein, changes the area occupied by the Prestin molecule within the plane of the membrane (Oliver et al. 2001; Dallos and Fakler 2002; Song and Santos-Sacchi 2013). When many Prestin molecules are clustered in the baso-lateral membrane and change shape at the same time, the outer hair cell length changes (Dallos et al. 1993; Iwasa 2001). An increase in cilia membrane tension in response to decreases in sound intensity would be an analogous function in *D. melanogaster*. We found evidence for specific *dpres* expression in the auditory sensory neurons. However, our study with flies lacking *dpres* found that *dpres* is dispensable for fly hearing.

### The anion antiporter activity of *dpres*

The primordial function of mammalian Prestin as an anion transporter, rather than a motor, is further supported by Albert et al. (2007) who show that the zebrafish Prestin ortholog does not possess the same electromotility as mammalian Prestin, along with altered kinetics and voltage dependence. In fact, non-mammalian Prestin orthologs are electrogenic anion transporters that exchange monovalent versus divalent anions (Schaechinger and Oliver 2007; Deng et al. 2013). The explanation for lack of a *dpres* role in *D.melanogaster* hearing is supported by the studies of Okoruwa et al. (2008), who surveyed the electromotility of Prestin homologs among vertebrates, by looking at protein sequence conservation. They found that the electromotility appears to be a derived character that is distinctly mammalian, especially in eutherian mammals. They argue that two insertion/deletion events and subsequent evolution conferred fast electromotility in the transmembrane anion transporter domain of Prestin. While this divergence to electromotility in mammalian Prestin was thought at the time to coincide with anatomical innovations in the outer hair cells and surrounding cochlear tissue of eutherian lineage, namely the reduction in number of rows of IHCs and in OHCs and changes in the tectorial membrane structure, the inference of chick Prestin’s role in short hair cell amplification (Beurg et al. 2013) suggests that electromotile properties may have predated the mammalian anatomical arrangement.

Because the *dpres* gene has been functionally retained in the *D. melanogaster* genome, is expressed in sense organs at all major stages of its life cycle, and its product mediates robust anion transport (Fig. 5) (Hirata et al. 2012a, b), we expect that dpres acts as an anion transporter in the normal function of the neuron. If that is the case, one might still expect to find that *dpres* mutants have reduced hearing due to defects in functional neurons. A possible explanation for the lack of a quantifiable *dpres* mutant phenotype is the presence of other proteins that may serve a redundant function to dpres in the chordotonal organ. A search of genes differentially expressed in the JO (Senthilan et al. 2012) identified ten solute carriers, though none closely related to dpres. The possibility remains, though, that one of these transporter genes serves a redundant function throughout the mechanosensory tissues in which it is expressed and can thus compensate for the loss of *dpres* function, or that functionally equivalent solute carriers replacing dpres are not selectively expressed in the mechanosensory organ. Alternatively, it is possible that functional consequences of *dpres* mutation in the JO can only be revealed under certain conditions that differ from the standard laboratory environment, such as at the fringes of tolerable temperature or under conditions of cell stress.

### Are acquisition of Prestin electromotility and loss of *nompC* coeval?

**Fig. 6:**
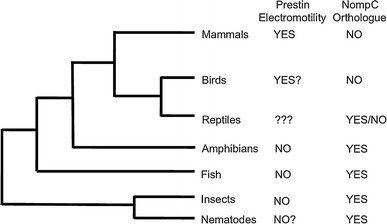
Evolution of Prestin-mediated somatic electromotility coincides with disappearance of NompC/TRPN1. Data from published work and Blastp searches of the representative protein databases derived from model organism genomes show that Prestin somatic electromotility coincides with the absences of *nompC* orthologs. Representative genomes available from NCBI were blasted with the *D. melanogaster* NompC isoform AAF52248.3 to identify whether there were *nompC* orthologs. Only those TRP channels with both the transmembrane and N-terminal ankyrin repeat structure representative of NompC were considered orthologous proteins. *Gallus gallus* and *Anas platyrhynchos* were used as representative birds. Among reptiles, the *Anolis carolinensis* genome appears to be devoid of *nompC* homologs, while *Chrysemys picta, Chelonia mydas, Pelodiscus sinensis, Alligator mississippiensis,* and *Alligator sinensis* each had recognizable *nompC* homologs. *Xenopus tropicalis* represented amphibians (Tang et al. 2013). The *Caenorhabditis elegans* *nompC* ortholog *Trp*-*4* was used as the basis for this figure (Kang et al. 2010). Information on Prestin electromotility from mammals, birds, reptiles, amphibians and fish was taken from published work (Dallos and Fakler 2002; Albert et al. 2007; Beurg et al. 2013; Tang et al. 2013). *Question marks* denote those organisms that have Prestin homologs with no (*C. elegans*) or conflicting (birds and lizards) data regarding putative somatic electromotility (Stewart and Hudspeth 2000; Tang et al. 2013)

An interesting evolutionary relationship may occur between Prestin and the gene *no mechanoreceptor potential C* (*nompC* or TRPN1). NompC is a mechanotransduction channel (Yan et al. 2013) and integral component of the *D. melanogaster* auditory mechanotransducer complex (Effertz et al. 2012) and has been shown to be essential for mechanical amplification in *D. melanogaster* hearing (Göpfert et al. 2006; Effertz et al. 2011). *nompC* homologs also are expressed in zebrafish (Albert et al. 2007) and *Xenopus tropicalis* (Shin et al. 2005), indicating widespread occurrence in lower vertebrates. Meanwhile, and in accordance with prior attempts (Delmas and Coste 2013), we have been unable to identify a mammalian or avian *nompC* ortholog. The search was conducted through Blastp using the full-length NompC isoform AAF52248.3 against the refseq protein database of representative mammals and birds (Fig. 6). In this search we defined an ortholog as having both the N-terminal ankyrin repeats and the C-terminal transmembrane domain.

Prestin’s derived cochlear amplifier function meanwhile has been reported to have evolved in both avian and mammalian lineages—lineages that do not contain *nompC* orthologs, while its sole (ancestral) function as an anion transporter is present in those species that have retained NompC as a functional mechanotransduction channel (Fig. 6). The association of Prestin’s derived function with loss of the *nompC* gene suggests an interesting hypothesis in which the non-linear sound amplification function of NompC has been replaced by Prestin. This replacement may be facilitated by the fact that *nompC* mutations in *D. melanogaster* reduce, but do not eliminate, auditory function (Eberl et al. 2000; Effertz et al. 2011). We are excited to see further functional and evolutionary analysis on both Prestin and *nompC* to test if this is an accurate reflection of the evolution of auditory sound amplification. Key progress in confirming an association between Prestin electromotility and loss of NompC will be made by analysis of reptiles, among which NompC homologs can be found in turtles and alligators, but so far not in lizards (Fig. 6). Reports of active amplification mechanisms in reptiles (Stewart and Hudspeth 2000) have yet to be linked to, or distinguished from, Prestin-based mechanisms (Fig. 6).

## Electronic supplementary material

Below is the link to the electronic supplementary material.

Supplementary material 1 Fig. S1: Alignment of dPres with human Prestin amino acid sequence. The amino acid sequences of dPres (AAF49285.1) and human Prestin (AAP31417.1) were aligned using Clustal Omega (http://www.ebi.ac.uk/Tools/msa/clustalo/) (Sievers et al. 2011). The ion transporter domain is underlined. Identical residues are shown by (*) while conserved substitutions are shown by (:) and semi-conserved substitutions are shown by (.). Red - small hydrophobic residues, blue– acidic residues, magenta– basic residues, green - hydroxyl + amine + basic + Q. The locations of engineered stop codons are highlighted in gray. (PDF 44 kb)

## Abbreviations

BM: Basilar membrane
FFT: Fast Fourier transform
IHC: Inner hair cell
JO: Johnston’s organ
OHC: Outer hair cell
PCR: Polymerase chain reaction
SEP: Sound-evoked potential
RT: Reverse transcriptase
